# Effect of Fluorohydroxyapatite on Biological and Physical Properties of MTA Angelus

**DOI:** 10.1155/2023/7532898

**Published:** 2023-11-06

**Authors:** Behnam Bolhari, Nazanin Chitsaz, Sara Nazari, Marjan Behroozibakhsh, Aidin Sooratgar, Atieh Hashemian

**Affiliations:** ^1^Department of Endodontics, School of Dentistry, Tehran University of Medical Sciences (TUMS), Tehran, Iran; ^2^Department of Dental Biomaterials, School of Dentistry, Shahid Beheshti University of Medical Sciences, Tehran, Iran; ^3^Department of Dental Biomaterials, School of Dentistry, Tehran University of Medical Sciences (TUMS), Tehran, Iran; ^4^Department of Endodontics, Tehran University of Medical Sciences (TUMS), International Campus, Tehran, Iran

## Abstract

**Objectives:**

This study aimed to assess the effect of addition of fluorohydroxyapatite (FHI) on biological and physical properties of mineral trioxide aggregate (MTA) Angelus.

**Materials and Methods:**

In this in vitro, experimental study, nano-FHI powder was first synthesized, and the morphology and chemical structure of particles were evaluated by scanning electron microscopy (SEM), Fourier-transform infrared spectroscopy (FTIR), and X-ray diffraction (XRD). Three groups were evaluated in this study: MTA Angelus, MTA modified with 10% FHA, and MTA modified with 15% FHA. After mixing, the materials were applied to ring molds (10 mm diameter, 1 mm height), and the setting time of the three groups was evaluated according to ISO6876 and ASTMC266-03 with a Gillmore needle. The pH was measured using a pH meter at 24 and 48 hours and 7 days after mixing. The cytotoxicity of the materials was assessed in freshly mixed form and after 1 and 7 days using the methyl thiazolyl tetrazolium (MTT) assay according to ISO10993-5. Data were analyzed by one-way and repeated measures ANOVA and Tukey's test (alpha = 0.05).

**Results:**

The addition of FHA to MTA significantly decreased the initial setting time (*P* < 0.05) and had no significant effect on cell viability (compared with pure MTA Angelus) at 1 and 7 days. However, modified MTA groups in freshly mixed form showed significantly lower cell viability (*P* < 0.05). The pH remained alkaline at all time points.

**Conclusion:**

Addition of 15% FHA to MTA Angelus decreased its setting time with no adverse effect on cell viability (except for fresh form) or pH.

## 1. Introduction

The ultimate goal of root canal therapy is to seal the communication between the root canal space and periapical tissue to prevent leakage of infective agents and microorganisms [1]. The success of endodontic treatment depends on several factors, particularly three-dimensional apical and coronal seals [2]. Moreover, the materials used to create an optimal seal must be biocompatible, nontoxic, noncarcinogenic, and radiopaque, with optimal dimensional stability, antibacterial properties, and handling properties. Also, they must have favorable physical characteristics such as high compressive strength, bond strength to dentinal walls, and hardness [3].

Mineral trioxide aggregate (MTA) is currently one of the most commonly used dental materials. It is composed of tricalcium silicate, dicalcium silicate, tricalcium aluminate, tetracalcium aluminoferrite, calcium sulfate, and bismuth oxide [4]. MTA was first introduced for perforation repair [5] and as a root-end filling material [6]. However, it currently has extensive applications for vital pulp therapy as a pulp capping agent [7], pulpotomy [8], root perforation repair [9], creation of an apical seal in nonvital, open-apex teeth [10], and regenerative endodontic treatments [11]. Evidence shows that MTA has high biocompatibility [12] and low cytotoxicity [13] compared with other commonly used endodontic materials. It also elicits an optimal tissue response [14]. Its application as a root-end filling material decreases periapical inflammation and induces the formation of a new cementum layer in contact with the cement [15]. MTA has high sealing ability [16], favorable marginal adaptation [17], and optimal antimicrobial activity [18].

Portland cement, which is a hydraulic cement, is the main constituent of MTA [5]. The hydration process of hydraulic cement is initiated after the contact of powder with water and leads to the formation of a porous gel. The setting reaction of MTA takes several days. As the hydration process is accomplished, porosities decrease and resistance increases [19].

MTA has some shortcomings such as tooth discoloration, long setting time [20], and difficult handling due to its consistency after mixing the powder with water [21]. High cost is another drawback of MTA, which limits its extensive application by dental clinicians [22]. However, the manufacturer of MTA Angelus claims that it has a setting time of 15 minutes.

Attempts have been made to fix the drawbacks of MTA, and several materials were added to MTA powder to improve its physical, chemical, mechanical, and biological properties. Calcium chloride, K-Y gel, NaOCl gel, chlorhexidine gluconate, propylene glycol, aluminum fluoride, nanosilica, and methyl cellulose were added to MTA, and the physical, chemical, mechanical, and biological properties of the obtained product were studied [21, 23, 24]. However, each material had a positive effect on one and a negative effect on another property of MTA, and thus, none of them appeared to be ideal for incorporation in the composition of MTA powder.

Hydroxyapatite (HA) is a calcium phosphate compound with a morphology and composition similar to human hard tissue. It has a hexagonal structure with chemical formula Ca_10_(PO_4_)_6_(OH)_2_ [25]. HA is biocompatible, bioactive, osteoconductive, and nontoxic [26]. It has been reported that addition of artificially synthesized nano-HA and fluorapatite (FA) to glass ionomers improves their mechanical properties and bond strength to dentin. Another study showed that the addition of HA and fluorohydroxyapatite (FHA) to epoxy resin-based endodontic sealers increased their microhardness and bioactivity [27]. Nano-FHA is a potential additive that can be used to improve the quality of dental materials [28]. Shafaee et al. [29] added nano-FHA to Essix retainer to prevent the development of dental caries, and Wang et al. [30] demonstrated that the addition of nano-FHA to poly-ether ether ketone dental implants enhanced their biocompatibility, antimicrobial activity, and bone attachment. Another study used FHA to enhance the mechanical strength of a glass ionomer [28]. Also, Lin et al. [31] indicated that the addition of nano-FA and nano-FHA to resin-modified glass ionomer enhanced fluoride release with no adverse effect on bond strength.

To the best of the authors' knowledge, no previous study has assessed the effect of the addition of FHA to MTA. Thus, this study aimed to assess the effect of the addition of FHA on biological and physical properties of MTA Angelus.

The null hypothesis of this study was that FHA could improve biological and physical properties of MTA Angelus.

## 2. Materials and Methods

This in vitro, experimental study evaluated the setting time, pH, and cytotoxicity of MTA Angelus (Angelus, Londrina, PR, Brazil) modified with 10% and 15% FHA by weight. The surface morphology and chemical composition of specimens were also evaluated by scanning electron microscopy (SEM), Fourier-transform infrared spectroscopy (FTIR), and X-ray diffraction (XRD).

The number of samples in each experiment was three.

### 2.1. Synthesis of FHA [32]

Calcium nitrate 4 H_2_O with 0.3 molarity was synthesized and transferred into a magnetic stirrer. The pH of the material was constantly monitored by a pH meter (WTW, Germany); 1 M sodium hydroxide was added to the solution until pH was stabilized at 10-11. Next, 0.18 M solution of ammonium dihydrogen phosphate and 0.18 M sodium fluoride were added to the calcium nitrate solution in a dropwise manner. The pH remained at 10-11 during the process of addition of NaOH. To separate FHA, the obtained solution was filtered and centrifuged several times. The obtained gel was rinsed with water and acetone and freeze-dried. Dry powder was sintered in a furnace at 600°C for 6 hours. The rate of temperature rise was 2°C/minute.

The study groups were as follows:

  Group 1 (control group): Unmodified MTA Angelus
  Group 2: MTA Angelus modified with 10% FHA [25]
  Group 3: MTA Angelus modified with 15% FHA [25]

### 2.2. Synthesis of FHA-Modified MTA

To synthesize the experimental groups (MTA modified with 10% and 15% FHA), MTA powder was mixed with 10 wt% and 15 wt% FHA as follows.

Nano-FHA and MTA powders were weighed by a digital scale and divided into equal portions. Each portion of nano-FHA powder (10 wt% of the final powder in group 2 and 15 wt% in group 3) was manually mixed with MTA Angelus powder for 3 minutes. Subsequently, they were mixed in an amalgamator to achieve homogenous distribution of particles.

### 2.3. Cytotoxicity

Cytotoxicity of the three groups was evaluated according to ISO10993-5 in freshly mixed form and also at 1 and 7 days after setting.

The powder and liquid were mixed according to the manufacturer's instructions in all groups, and the mixture was applied in molds measuring 5 mm in diameter and 2 mm in thickness to fabricate disc-shaped specimens for the three groups. At the respective time points, the specimens were removed from the incubator and also easily from the mold (since split molds were used). A serum-free culture medium was used for cytotoxicity testing. The specimens were incubated in 650 *µ*L of serum-free Dulbecco's modified Eagle's medium at 37°C, which yielded 1 mL/3 cm^2^ extraction ratio. The specimens were then agitated at a rate of 100 rpm in an orbital shaker (Stuart Scientific, Stone, UK). After 24 hours, the specimens were transferred to fresh culture medium and incubated for another 6 days. Accordingly, the cytotoxicity of the extract was evaluated at 1 and 7 days.

The cytotoxicity of the materials was evaluated by the methyl thiazolyl tetrazolium (MTT) assay. Fresh MTT was diluted in 0.5 mg/mL phosphate buffered saline. The obtained solution was filtered by sterile syringe filters and used on the same day.

Dental pulp cells (291 w) were purchased from the Iranian Genetic Resources, seeded in 96-well plates, and incubated in culture medium for 24 hours to double in count and create a semiconfluent monolayer. They were then subjected to the test materials in 10% and 15% concentrations. After 24 hours, the treated cells were incubated with MTT solution (0.5 mg/mL in phosphate buffered saline) at 37°C and 5% CO_2_ with over 90% humidity. After 3-4 hours of incubation, 100 *µ*L of dimethyl sulfoxide was added to dissolve the formed formazan crystals. The optical density of the solution was read after 20 minutes at 570 nm wavelength with 630 nm reference wavelength using a multiwell plate reader and compared with control cultures to calculate the percentage of cell viability. All the experiments were performed in aseptic conditions under a laminar flow cabinet. Plain culture medium and distilled water were used as positive and negative controls, respectively.

### 2.4. Setting Time

The setting time of specimens in the three groups was measured according to ISO6876: 2012 and ASTM C266-03 under controlled temperature and moisture (37 ± 1°C temperature and 95 ± 5% relative humidity). The samples were mixed according to the manufacturer's instructions and applied in stainless steel ring molds with 10 mm diameter and 1 mm height. Excess material was removed to obtain a smooth surface. Prior to testing, all instruments and materials, glass slab, and spatula were incubated at 23 ± 1°C temperature for one hour. The mold was incubated at 37 ± 1°C and 95% relative humidity for 24 hours.

To measure the initial setting time, a Gillmore needle with 100 ± 0.5 g weight and a cylindrical needle tip diameter of 2 mm with 5 mm length was used. According to the standards, 120 ± 10 seconds after completion of mixing in the mold, the mixture was incubated at 37 ± 1°C with 95% relative humidity. The Gillmore needle was gently pressed against the horizontal material surface for 5 seconds at 30-second intervals according to ISO6876: 2012. Any indentation on the surface indicated that the material had yet to completely set. This process was repeated until no indentation was created on the surface. This time was recorded as the initial setting time. The initial setting time was recorded for all three groups as explained above.

The final setting time was evaluated using a Gillmore needle with 456 ± 0.5 g weight with a flat end with 1.0 ± 0.1 mm diameter. The needle was gently pressed against the surface at 60-second intervals until a complete round-shaped indentation was no longer seen. This time was recorded as the final setting time.

### 2.5. Assessment of pH

The powder and liquid were mixed on a glass slab according to the manufacturer's instructions and applied in metal molds to create discs with 10 mm diameter. They were then incubated at 37°C and 100% humidity. After setting, the cements were removed from the molds and placed in sealed plastic containers filled with 5 mL of distilled water and incubated at 37°C and 100% humidity for 7 days. The pH of the stored solution was measured using a pH meter (Metrohm AG, 781 pH/Ion Meter; Switzerland) at 24 and 48 hours and 7 days after mixing. The pH of distilled water without cement was recorded as control.

#### 2.5.1. XRD Analysis

All synthesized powders underwent XRD analysis in an X-ray diffractometer (X'Pert PRO MPD, Panalytical Company, Netherlands) at 40 kV voltage and 40 mA amperage using Cu-Ka (1.5406 Å). For qualitative analysis, XRD graphs were drawn in the range of 10 ≤ 2*θ* ≤ 70 with a scanning speed of 0.02 s^−1^ with 0.026 step size.

#### 2.5.2. FTIR

The specimens underwent FTIR using Spectrum Two (PerkinElmer, USA) at 350–8300 cm^−1^ with 0.5 cm^−1^ resolution.

#### 2.5.3. SEM

The specimens were gold sputter-coated (EMITECH K450X, England), and their surface microstructure was inspected under a scanning electron microscope (Sigma VP, ZEISS, Germany) at 10 kV.

### 2.6. Statistical Analysis

Data were analyzed by one-way and repeated measures ANOVA. Pairwise comparisons were performed using Tukey's test at *P* < 0.05 level of significance.

## 3. Results

### 3.1. MTT Assay Results for Cytotoxicity

Table 1 presents the mean optical density of the three cements in freshly mixed form and 1 and 7 days after setting. As shown, the addition of FHA to MTA decreased cell viability in both groups at all time points except for day 1 in the 10% FHA group. According to one-way ANOVA, a significant difference was noted in cell viability of the three groups in fresh form (*P* = 0.00), but the difference was not significant at 1 or 7 days (*P* > 0.05). Pairwise comparisons of the cell viability of the three groups in fresh form were then performed using Tukey's test (Table 2). The results showed that cell viability in the control and MTA groups was significantly higher than that in 10% FHA and 15% FHA groups (*P* < 0.05). No other significant differences were noted (*P* > 0.05). Within-group comparison of cell viability over time revealed a similar trend in all groups. In all three groups, cell viability in fresh form was significantly lower than that after 1 and 7 days (*P* < 0.05), but the difference in cell viability between 1 and 7 days was not significant in any group (*P* > 0.05).

### 3.2. Setting Time

Table 3 presents the mean initial and final setting times in the three groups. One-way ANOVA revealed a significant difference in initial setting time among the three groups, such that the initial setting time in the 15% FHA group was significantly shorter than that in the 10% FHA group (*P* < 0.05). Also, the initial setting time of the 10% FHA group was significantly shorter than that of the MTA group (*P* < 0.05).

The final setting time of the 15% FHA group was significantly shorter than that of 10% FHA and MTA groups (*P* < 0.05); the difference between 10% FHA and MTA was not significant (*P* > 0.05).

### 3.3. Assessment of pH

Table 4 presents the pH in the three groups at different time points. A significant difference was noted in pH among the three groups at each time point (*P* < 0.05). At 24 hours, the pH order was as follows: MTA > 10% FHA > 15% FHA. At 48 hours, the pH order was as follows: 10% FHA > MTA > 15% FHA. At 7 days, the following order was recorded: 15% FHA > MTA > 10% FHA. The change in pH values at different time points within each group was not significant (*P* > 0.05).

#### 3.3.1. XRD Results

**Figure 1:**
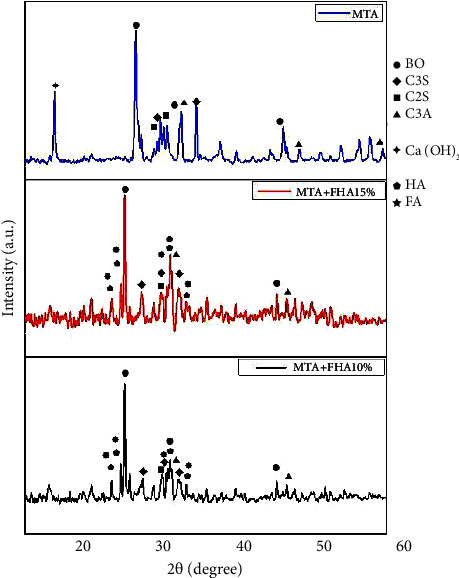
XRD graph of the three tested cements. Dominant crystals in each group are shown (BO, bismuth oxide; C3S, tricalcium silicate; C2S, dicalcium silicate; C3A, tricalcium aluminate; Ca(OH)_2_, calcium hydroxide; HA, hydroxyapatite; FA, fluorapatite).

Figure 1 presents the XRD graph of the three tested cements. As shown, HA and FA were found in groups containing FHA. Tricalcium silicate, dicalcium silicate, tricalcium aluminate, and bismuth oxide were identified in all groups.

XRD analysis of MTA Angelus showed a peak at 18° 2-theta for calcium hydroxide according to ICDD 01-076-0571. Considering the fact that bismuth oxide, tricalcium silicate, and tricalcium aluminate are present in the composition of MTA, the peaks related to these compounds were seen in all three groups as shown in Figure 1. Bismuth oxide and tricalcium silicate peaks were noted at 28°. In groups 2 and 3 (FHA-modified MTA), since no specific pattern has been defined for FHA, assessment was performed according to FA (JCPDS card #15–0876) pattern. The peaks at 26°, 32°, and 33° belonged to the apatite compound. Such peaks were also noted in the MTA group due to its composition. However, in the apatite-containing groups, the apatite peaks merged with the peaks related to the MTA constituents, and thus, the intensity of the peaks and a shift to right were noted at 32° and 33°, indicating the presence of apatite in the compound. The peak at 26° confirmed the presence of apatite. No significant difference was noted between groups 2 and 3 due to different percentages of FHA.

#### 3.3.2. FTIR

**Figure 2:**
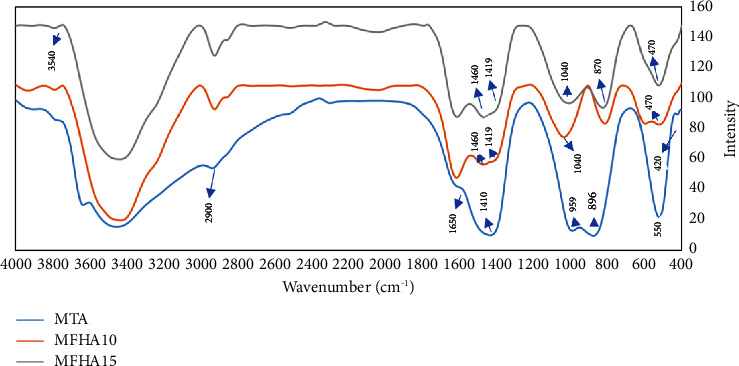
FTIR spectra of the three groups.

Figure 2 presents the FTIR spectra of the three groups. As shown, in MTA Angelus, absorption bands were noted at 1410 cm^−1^ and 896 cm^−1^ related to the C-O group. The band at 2900 cm^−1^ belonged to the -CH group, which becomes prominent in hydrated (set) materials [32]. Also, a wide peak was seen at 3000–3600 cm^−1^ attributed to the water OH group present in the compound. The bands at 420, 550, and 959 cm^−1^ were related to calcium silicate hydrate (C-S-H). A peak at 1410 cm^−1^ related to carbonate (CO_3_) was also seen.

In FHA groups, the peaks at 470, 870, and 1040 cm^−1^ were related to vibrations of *ν*_2_ (P-O, *v*_1_(*P* − *O*) and *v*_3_ (*P* − *O*).). Also, vibrations of *v*_2_ (*C* − *O* 870 cm^−1^) and *v*_3_ (*C* − *O* 1419 − 1460 cm^−1^) were seen in FHA groups. In FHA, the apatite structure was noted at 560–610 and 950–1100 cm^−1^. The peak at 1040 cm^−1^ was related to the phosphate bond in the structure of apatite, which was seen in both FHA groups. The peaks at 1040 cm^−1^ and with a slight shift at 870 cm^−1^ in the 15% FHA group were longer and more distinct than those in the 10% FHA group. A new band (OH-F or OH-F-HO) was noted at 3540 cm^−1^ in FHA groups.

#### 3.3.3. SEM

**Figure 3:**
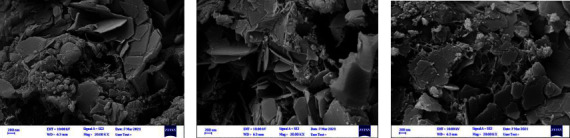
SEM micrographs of the completely set specimens in the three groups: (a) MTA Angelus; (b) 10% FHA; (c) 15% FHA.

Figure 3 presents the SEM micrographs of the completely set specimens in the three groups. SEM micrographs of all three groups showed apatite crystals in the form of plates with sharp margins along with spherical agglomerated particles. It appears that the addition of FHA slightly altered the morphology of crystals and increased their aspect ratio. The addition of FHA increased the accumulation of plate-like particles, especially in the 15% FHA group.

## 4. Discussion

This study assessed the effect of the addition of 10% and 15% FHA on biological and physical properties of MTA Angelus. The 10% and 15% values by weight for the addition of FHA were selected according to a previous study that showed lower concentrations did not add any advantage to MTA and higher concentrations complicated the manipulation and handling of MTA [25]. The present results revealed that 15% FHA had the shortest initial and final setting time. Also, the initial setting time of the 10% FHA group was shorter than that of MTA, but its final setting time was comparable to that of MTA with no significant difference. This finding was in line with the results of Antonijevic et al. [33] who showed that the addition of nano-HA to calcium silicate-based cement significantly decreased the initial setting time. However, Guerreiro-Tanomaru et al. [34] found that the addition of nano-HA to calcium silicate-based cement had no significant effect on initial setting time but decreased the final setting time. Eskandarinezhad et al. [35] used HA to shorten the setting time of MTA. Both HA and FHA nanoparticles are highly active due to their nanostructure and form bonds faster; thus, they shorten the setting time of MTA [35].

The present results revealed that the pH of MTA Angelus at 24 hours was higher than that of the other groups. At 48 hours, the 10% FHA group showed a higher pH, while at 7 days, the pH of the 15% FHA group was higher. Although a significant difference existed in the pH of the three groups at each time point, the difference was not clinically important, and the pH values of all groups were in the alkaline range at different time points. Antonijevic et al. [33] and Guerreiro-Tanomaru et al. [34] reported that addition of nano-HA to calcium silicate-based cement did not change pH. Similar to the present study, all values were within the alkaline range. Although they added nano-HA instead of FHA, the alkaline pH of cements may biologically induce mineralization [36]. Alkaline pH can cause inflammatory reactions and formation of hydroxyapatite [37] and reparative dentin [38]. Release of calcium ions from the MTA cements and increased alkalization of the surrounding environment are related to their ability to produce calcium hydroxide, which reacts with water and releases calcium and hydroxyl ions. The released hydroxyl ions are responsible for the pH rise in the surrounding environment [39].

The present results indicated that the addition of FHA to MTA did not change cell viability at 1 and 7 days, compared with MTA Angelus. However, in the fresh state, cell viability of FHA groups was lower than that of MTA Angelus. This result was in agreement with the findings of Liu et al. [40] who showed that the addition of FHA adversely affected the cell viability. Tahriri and Moztarzadeh [41] and Montazeri and Shokrgozar [42] demonstrated that the addition of FHA did not increase cytotoxicity, which was different from the current results obtained in fresh form of cements. Since all studies used the MTT assay for cytotoxicity testing, variations in the results may be due to the use of different cell types. For instance, Liu et al. [40] used osteoclasts and Tahriri and Moztarzadeh [41] used G-292 cells and osteoblasts, while we used 291 w dental pulp cells, which are a type of mesenchymal stem cells. Montazeri and Shokrgozar [42] used osteoblasts and mesenchymal stem cells and showed that the presence of FHA increased the proliferation of osteoblasts but had no significant effect on mesenchymal stem cells. The presence of fluoride in the composition of FHA increases the release of alkaline phosphatase in osteoblasts; however, this is not the case for mesenchymal stem cells. Also, it appears that the fluoride ions in the structure of FHA affect the release of calcium and phosphate ions and the subsequent biological response. Thus, the selection of the most appropriate concentration for an optimal biological response is highly important [42].

In the present study, the XRD analysis showed that the XRD pattern of FHA was somewhere between that of HA and FA. Due to high similarity in the crystalline structure and the same size of network, all peaks of FA and HA could not be precisely differentiated. Widening of the calcium hydroxide peak indicated a reduction of the crystalline phase and its conversion to an amorphous phase, which cannot be identified by XRD [23].

Rocha et al. [43] disclosed the FTIR spectra of MTA Angelus and showed absorption bands at 660, 896, and 1410 cm^−1^ related to C-O. In the present study, absorption bands were noted at 896 and 1410 cm^−1^ for MTA Angelus. Abu Zeid et al. [44] showed bands at 2800–2900 cm^−1^ related to the -CH group, which were found at 2900 cm^−1^ for MTA Angelus in the present study. The formation of CO_3_ after setting of material results in the formation of peaks at 1440 and 1472 cm^−1^, which were seen at 1410 cm^−1^ for MTA Angelus in the present study. Bands at 449, 524, and 996 cm^−1^ are related to calcium silicate hydrate in MTA Angelus, which were seen at 420, 550, and 959 cm^−1^ for the MTA Angelus used in the present study. An extensive peak at 3000–3600 cm^−1^ was attributed to the water OH group in completely set materials in the three groups, indicating the formation of hydrated phases (such as calcium hydroxide and calcium silicate hydrate) as reaction products. Even in case of presence of free calcium hydroxide as a reaction product, some additives can react with it and decrease its content. In the present study, the absence of calcium hydroxide in set MTA may indicate its reaction with other groups.

In FTIR of FHA groups, phosphate and carbonate functional groups were observed. The presence of carbonate in all three study groups may be due to the absorption of carbon dioxide from the atmosphere, and the phosphate band was related to the apatite structure with a longer and more distinct peak in the 15% FHA group [45]. A peak at 3540 cm^−1^ related to hydroxyl groups was noted as well. The presence of hydroxyl groups was due to the hydrogen bond of fluorine ions. This peak confirmed the crystalline structure of FA with the hydroxyl group. These observations are due to the formation of hydrogen bonds between F and OH, which indicates that F ions have replaced OH ions in the structure of apatite. Nonetheless, the exact amount of F ions replaced with OH ions cannot be determined by FTIR [46].

The microstructure of FHA is plate-like with different aspect ratios. Layered patterns have been seen in FHA specimens. Researchers claim that binding of F- ions affects the morphology of apatite crystals [47, 48]. In this study, the replacement of fluoride in the crystalline structure of apatite changed the morphology of crystals and increased their aspect ratio. Sharp margins of FHA crystals indicate their highly crystalline nature.

It has been reported that fluoride can cause epitaxial growth of crystals on the precursor of octa-calcium phosphate and change the morphology of apatite crystals as such [49]. Fan et al. [50] reported that fluoride has a dose-dependent effect on the morphology of calcium phosphate crystals [50].

Future studies are required on other percentages of FHA and their effects on other physical, mechanical, and biological properties of MTA. Further studies on the ionic and crystalline forms of the constituents of these cements are also recommended. In vivo studies are required to better simulate the clinical setting.

## 5. Conclusion

Addition of 15% FHA to MTA Angelus decreased its setting time with no adverse effect on cell viability (except for fresh form) or pH.

## Figures and Tables

**Table 1:** Mean optical density (indicative of cell viability) in the three cement groups in freshly mixed form and at 1 and 7 days after setting (*n* = 3).

Time	Groups	Mean	Std. deviation	*P* value
	Control	0.164	0.006	0.000
Fresh	MTA	0.153	0.005	
	MTA-10% FHA	0.139	0.004	
	MTA-15% FHA	0.135	0.001	

Day 1	MTA	0.184	0.007	0.785
	MTA-10% FHA	0.185	0.006	
	MTA-15% FHA	0.181	0.004	

Day 7	MTA	0.175	0.004	0.479
	MTA-10% FHA	0.172	0.002	
	MTA-15% FHA	0.172	0.004	

**Table 2:** Pairwise comparisons of cell viability of the three groups by Tukey's test.

1^st^ group	2^nd^ group	*P* value
Control	MTA	0.107
	MTA-10% FHA	0.001
	MTA-15% FHA	0.001

MTA	MTA-10% FHA	0.034
	MTA-15% FHA	0.010

MTA-10% FHA	MTA-15% FHA	0.811

**Table 3:** Mean initial and final setting time (in minutes) in the three groups (*n* = 3).

Group	Initial setting time (min)	Std. deviation	Final setting time (min)	Std. deviation
MTA	100	0.000	120	0.000
MTA-10% FHA	57.5	2.5	120	0.000
MTA-15% FHA	42.5	2.5	107.5	2.5

**Table 4:** pH in the three groups at different time points.

Time	Group	pH
24 hours	MTA	8.9
	MTA-10% FHA	8.85
	MTA-15% FHA	8.65

48 hours	MTA	8.6
	MTA-10% FHA	8.65
	MTA-15% FHA	8.55

7 days	MTA	8.1
	MTA-10% FHA	7.85
	MTA-15% FHA	8.4

## Data Availability

The data used to support the findings of this study are included within the article and are available from the corresponding author upon request.
